# Assessing surgical management of corneal perforation in brittle cornea

**DOI:** 10.6026/973206300211559

**Published:** 2025-06-30

**Authors:** Jyoti Mani Shankar Pandey

**Affiliations:** 1 Department of Ophthalmology, Nalanda Medical college and Hospital, Patna, Bihar, India

**Keywords:** surgical management, corneal perforation, brittle cornea

## Abstract

Evaluate a sample of patients who underwent surgical treatment for corneal perforation in brittle cornea by Penetrating keratoplasty and patch graft. In this study, 466 patients experiencing corneal perforation (CP) in brittle corneas that underwent surgery were retrospectively reviewed. They were divided into two categories. Category A: Patients received Penetrating keratoplasty (n= 232). Category B: Patients received patch graft (n=234) Following surgery, patients were evaluated on days one, seven, four, six, and 12 weeks. There was significant improvement in best corrected visual acuity (BCVA), uncorrected visual acuity (UCVA) in both Penetrating keratoplasty and Patch graft procedure for management of corneal perforation in brittle eyes. However, complications were lesser in patch graft method.

## Background:

Brittle cornea syndrome (BCS) is a rare connective tissue disorder marked by extreme corneal thinning and fragility, posing substantial surgical and diagnostic challenges in ophthalmology [1]. It is a rare autosomal recessive connective tissue disorder with ocular manifestations often associated with extraocular involvement; corneal thinning, blue sclera, keratoconus, keratoglobus, high myopia and irregular corneal astigmatism are identified [2, 3]. These structurally compromised corneas are highly susceptible to spontaneous or minor trauma-induced perforations, often resulting in a rapid loss of ocular integrity and vision [4, 5-6]. While previous studies have described the biomechanical and clinical characteristics of BCS, the management of acute perforation remains a significant concern, with limited standardized protocols. Empirical evidence demonstrates that suturing brittle corneas carries a high risk of extension of lacerations, wound dehiscence, and intraoperative rupture, necessitating meticulous surgical technique and preoperative planning [7, 8-9]. Delayed or inadequate intervention often leads to globe collapse or irreversible visual loss, emphasizing the urgency of early recognition and proper surgical strategy [10, 11-12]. The etiology of corneal perforation in BCS is multifactorial, with contributing factors including autoimmune keratitis, microbial infections, trauma, and epithelial dysfunction-all of which can exacerbate stromal degradation and perforation risk [13, 14-15]. Furthermore, defects in the epithelial barrier can promote chronic inflammation and sterile or infectious corneal melts, ultimately progressing to full-thickness perforation [16, 17, 18- 19].

Tectonic keratoplasty remains a cornerstone surgical approach to restore structural integrity and maintain ocular anatomy in these cases. Depending on perforation size, location, and stromal integrity, surgeons may choose between full-thickness penetrating keratoplasty, lamellar grafts, or corneal patch grafts. However, outcomes in brittle cornea patients following such procedures remain underreported. Therefore, it is of interest to critically evaluate clinical outcomes of tectonic surgical repair-specifically penetrating keratoplasty and patch grafting-in patients with brittle cornea syndrome who presented with spontaneous or traumatic corneal perforation. By retrospectively analyzing these cases, the study aims to contribute to the evolving surgical approach and postoperative management strategies in this high-risk population.

## Materials and Methods:

In this study Department of Ophthalmology Nalanda Medical College and Hospital, Patna, Bihar, India for one year. 466 patients experiencing CP in brittle corneas that underwent surgery were retrospectively reviewed. Population statistics, healthcare history, corrected-distance beforehand and subsequent Snellen visual acuity, specifics, surgical result and adverse effects, and ocular stability were among the data analyzed from medical documentation. Two surgical procedures were used to treat all patients with a confirmed CP who had a thorough ocular assessment: These two surgical techniques were penetrating keratoplasty and patch graft technique. Achieving the globe's structural integrity was the primary goal of the treatment. The dimensions and position of CP, the condition of the interior ocular tissues, and the presence of an intraocular contamination all influenced the surgical method that was chosen. Three groupings of sizes were identified: tiny (0.1-2.5 mm in diameter), medium (2.5-5.0 mm), and giant (diameter greater than 5.0 mm). The location was classified as peripheral (≥8.0mm, including the limbus), paracentral (zone exceeding 5.0mm up to 8.0mm), or central (including the visual axis, ≤5.0mm diameter of the central cornea). Corneoscleral patch grafting was typically used if the hole was minor or medium and there was no disease along the visual axis. Penetrating keratoplasty was the recommended surgical procedure for large perforations with extensive corneal melting or thinning, necrosis, and internal bulbar tissue damage.

Category A: Patients received Penetrating keratoplasty (n= 240)

Category B: Patients received patch graft (n=240)

Following surgery, patients were evaluated at baseline, four weeks, eight weeks and 12 weeks. After surgery, prednisolone acetate eye drops (1%) were applied eight times a day, and gatifloxacin eye drops (0.3%) were applied for four occasions a day. Additionally, mydriatic along with anti-glaucoma drugs were administered as needed. Over six weeks, prednisolone was lowered. Drug administration lasted for six weeks. At every visit, slit lamp results, best corrected visual acuity (BCVA), and uncorrected visual acuity (UCVA) were calculated in fractions by Snellens chart.

## Statistical analysis:

SPSS version 22.0 statistical analysis software was used to perform a statistical analysis of the data. Significance of difference (Kruskall-Wallis test), Chi-square test was used for comparing UCVA, BCVA in study groups at different follow ups. Mann-Whitney U test was used for comparisons of induced astigmatism in two incision groups.

## Results:

Males constituted 52% study population in Penetrating Keratoplasty category and 48% study population in Patch graft category (Table 1 - see PDF). There was improvement in mean UCVA at each visit in Penetrating keratoplasty group and the change at different visits was significant statistically (p=0.021). There was improvement in mean UCVA at each visit in Patch graft category and the change at different visits was significant statistically (p=0.011). At each visit, mean UCVA values were comparable in both Penetrating keratoplasty group and Patch graft group showing no significant variation between two categories (p>0.05) (Table 2 - see PDF). There was improvement in mean BCVA in Penetrating keratoplasty category and the change between baseline and final follow up was significant statistically (p=0.010). There was improvement in mean BCVA Patch graft category and the change between baseline and final follow up was significant statistically (p=0.001). At each visit, mean BCVA values were comparable in both Penetrating keratoplasty group and Patch graft group showing no significant variation between two categories (p>0.05) (Table 3 - see PDF). Complications were lesser in Patch graft category (22.64%) when compared against Penetrating keratoplasty group (29.31%). Mann-Whitney U test revealed statistically significant lesser complications in Patch graft category (Table 4 - see PDF).

## Discussion:

Corneal rupture is a common condition that can significantly impair vision and cause ocular morbidity. It can be caused by bacterial and non-infectious abnormalities of the ocular surface, trauma, immune system problems, and the spread of infection [23, 24-25]. Both primary and secondary epithelial defects contribute to corneal inflammation and eventual perforation [11, 12-13]. The aim of this study is to evaluate a sample of atients who underwent surgical treatment for corneal perforation in brittle cornea by Penetrating keratoplasty and patch graft. In our study, there was improvement in mean UCVA at each visit in Penetrating keratoplasty group and the change at different visits was significant statistically (p=0.021). There was improvement in mean UCVA at each visit in Patch graft category and the change at different visits was significant statistically (p=0.011).The findings of our study are similar to findings of other studies [23, 24, 25- 26]. These studies also found improvement in mean UCVA in patients having surgery for corneal perforation in brittle eyes by Penetrating keratoplasty surgery as well as Patch graft surgery [21, 22, 23-24]. After the initial surgical procedure provides temporary relief, a multistage surgical method is frequently employed as the last resort to restore visual function [14, 15-16]. A number of criteria influence the therapeutic process and when it should be carried out, including the etiology, the position and size of the perforation, corneal penetration and disintegration, and the condition of the internal globe tissues [12, 13, 14-15]. Brittle corneal holes can be surgically treated using tectonic keratoplasty. The preferred surgical techniques include full-thickness penetrating keratoplasty, anterior lamellar keratoplasty (ALK) and corneal patch grafts. There was improvement in mean BCVA Patch graft category and the change between baseline and final follow up was significant statistically (p=0.001). The observations of our study are supported by findings of other studies. These studies also found similar improvement in BCVA in surgical procedure of Penetrating keratoplasty and Patch graft for management of corneal perforation in brittle eyes [19, 20, 21, 22-23]. Brittle cornea condition is one of the few special cases in ophthalmology that is bothersome to the patient and the operating physician [10, 11-12]. In this instance, the globe's form and structural integrity cannot be maintained due to the thin and sensitive corneas' increased susceptibility to minor unintentional or unexpected corneal perforations [12, 13, 14-15]. In our study, complications was lesser in Patch graft category (22.64%) when compared against Penetrating keratoplasty group (29.31%). Brittle cornea syndrome is among the few special scenarios in ophthalmology that are a nightmare not only for the operating surgeon but also for the patient [23, 24, 25-26]. In order to save these corneas, it is essential to make the correct diagnosis, suture them successfully, and take the necessary precautions [21, 22-23]. Closing the corneal perforation and stitching a brittle cornea are very difficult procedures that require extreme caution and care [15, 16, 17- 18]. If the right precautions are not taken throughout the procedure, it may be difficult to save the eye.

## Conclusion:

There was significant improvement in BCVA, UCVA in both Penetrating keratoplasty and Patch graft procedure for management of corneal perforation in brittle eyes. However, complications were lesser in patch graft method.
